# Supplementary material for: Applications of statistical experimental designs to improve statistical inference in weed management
Source: PLoS One. 2021 Sep 15;16(9):e0257472. doi: 10.1371/journal.pone.0257472 (PMC8443065; doi:10.1371/journal.pone.0257472)

We greatly appreciate your review and comments. We responded and/or addressed each comment below. Thank you.

General Comment

In addition to the weed branch, a useful study has been written that will easily adapt to the agricultural field. The article is written from an innovative perspective. Although the study contains intensive information theoretically, there are doubts about its transfer to practice. The number of iterations (1000) of the simulation study was kept at an insufficient level. It is known in the literature that the results change with the increase in the number of simulations. Therefore, the number of iterations should be increased in the simulation.

Response 1: We agree that a larger number of iterations would provide more precise simulation results in any simulations. We had to consider computational time particularly for simulating the adaptive designs. We used 1,000 iterations in Sections 3.3, 4.4, and 5.3 due to the long computational time, and we believe that the patterns (superiority of the optimal design and of the adaptive design) are consistent and match with our intuition. In fact, we used 10,000 iterations in Section 2.2 because the computational time was relatively short in these simulations. We realize that we did not mention the number of iterations (10,000) in Section 2.2, so we added a sentence. Thank you. [Line 130]

The simulation steps can be made more understandable by showing them with a diagram.

Response 2: See Response 16.

The distributions used in the simulation study do not include all the conditions in terms of the shape of the distribution. Therefore, especially t(10), t(5), β(5,10), β(10,5) and χ2(3) distributions should be included in the study.

Response 3: See Response 15.

In addition, while generating random numbers, they should be produced and reported by taking into account different effect sizes.

Response 4: We believe that this comment is associated with the simulation results in Section 2.2 and Fig 1. We agree that the effect size is theoretically well defined for comparing two groups, and it has an important implication from the perspective of statistical theory. Our primary point, however, is to demonstrate that the superiority of the adaptive design (over the balanced design) as sigma2 deviates more from sigma1. In this interdisciplinary journal, we believe that the current version of demonstration will benefit the readers more than a heavy-theoretically-geared demonstration. We hope you understand our intention. Thank you.

As a result of the study, could different criteria be used besides Bias, Variance, and MSE arguments? (Table 1 and Table 2). So, are these criteria a sufficient argument for comparing the results?. Comparing the power (β) and type I error rates (α) of each of the tests in the scenarios createdwill strengthen the study.

Response 5: In Section 2.2, we used the probability of rejecting the null hypothesis (power or Type I error rate depending on the true scenario) because the objective in the section is hypothesis testing. In Section 3.3 (Table 1) and Section 4.4 (Table 2), the objective is parameter estimation, not hypothesis testing. It is very common to use the bias, variance, and MSE in the problem of parameter estimation. Of course, there are other criteria for evaluating estimation like penalizing underestimation more than overestimation and visa versa. However, it is not a scope of our primary focus in this manuscript. If it is of interest, we believethat it should be addressed via a separate paper.

Which packages of the R program were used for the simulations?. If the package is not used and a new function is written, it should be stated in the manuscript.

Response 6: All computational work was done by writing codes with built-in functions in R Version 4.0.2. It is stated in the revised manuscript. Thank you. [Lines 154-155, 263-265]

Specific comments

Abstract

This part is written very generally and the material of the study, the methods applied and the results should be mentioned. The author needs to re-write this and improve novelty.

Response 7: The entire abstract is rewritten more specifically. In the revised abstract, we clearly stated that we used simulations (a method to demonstrate the outperformance of the experimental designs) and summarized the results of the simulations (reduced error in parameter estimation and increased statistical power in hypothesis testing). [Abstract before Line 1]

Introduction

Line 16 – 17, The criticism made for Duncan multiple comparison does not reflect the truth. In recent simulation studies, Duncan's superiority over other methods is understood. These statements should be supported by the literature.

Response 8: It has been known that Duncan's multiple comparison inflates the Type I error rate as the number of treatments increases. We supported the statement by adding a reference (Petrinovich & Hardyck, 1969). [Line 16] If Duncan's multiple comparison is superior over the other methods (higher statistical power), it must be due to the inflated Type I error rate which is not a fair comparison.

Line 22, the sentences are not flowing well in paragraph entry. Please rephrase

Response 9: Thank you for your suggestion. We rephased and reordered some sentences, and we removed an unnecessary sentence. [Lines 22-32]

Line 22 and 25, These statements should be supported by the literature.

Response 10: To support the statement about the violation of homogeneity assumption, we added a reference (Lix, Keselman & Keselman, 1996). [Lines 23] The statement about the case of homogeneity assumption can be proven by calculus, so we added a phrase "it can be shown by calculus that …" [Lines 25-27]

Line 30 and 33, These statements should be supported by the literature.

Response 11: We added two references (Benjamini & Braun, 2002; Kramer, 1956)

Line 43 and 44, First, why wasn't the sample size determined using power analysis instead? Second, instead of dividing the dataset and estimating group variances, why not crossvalidation by dividing the dataset into testing and training?

Response 12: Regarding the first point, researchers have difficulties specifying a realistic effect size. Furthermore, due to logistics (e.g., fixed budget, maximally available sample size), it is not always possible to obtain a desirable sample size. Instead, given a maximally available sample size, it would be a more practical suggestion to consider an optimal design (i.e., distribute the fixed sample size effectively). This point is added in the paragraph. [Lines 37-39] Regarding the second point, cross-validation is a method which is devised for prediction not for hypothesis testing. In the two-sample hypothesis testing, minimization of the standard error is the most straightforward approach.

The differences between the two-stage design and the bayesian approach should be clearly stated.

Response 13: It is difficult to state the difference between the two-stage design and the Bayesian approach. The Bayesian approach (prior knowledge and updated knowledge about a parameter of interest) is used to make a decision (design an experiment) at each stage of experiment. [Lines 96-97]

Materials and Methods and Results

This section is difficult to follow especially the results, are not well structured and clearly reported.

Response 14: This manuscript does not have a single section for Materials and Methods and a single section for Results. In each section (Sections 2 to 5), there are results from the simulations (Sections 2.2, 3.3, 4.4, and 5.3) and results from the experiments by real data (Section 4.3).

Only the normal distribution was used in the simulations for two-group comparisons. It is known that the shape of the distribution affects the power of the test. Therefore, the distributions N(0,1), t(10), t(5), β(5,10), β(10,5) and χ2(3) should be used.

Response 15: Thank you for your suggestion. To match the mean and standard deviation of each simulation scenario, the suggested distributions were standardized, scaled by the standard deviation, then shifted by the mean. See Figure A at the end of this document. The overall pattern (superiority of the adaptive design over the balanced design) is maintained under the violations of the normality assumption. The adaptive design showed a higher Type I error rate (about 0.08) when data were generated under $\chi^2(3)$ and scaled by $\sigma_1 = \sigma_2 = 10$, and the balanced design showed a higher Type I error rate (about 0.07) when data were generated under $\chi^2(3)$ and scaled by $\sigma_1 = 10$ and $\sigma_2 = 100$. We briefly summarized this result at the end of Section 2.2. [Lines 160-168] There is no point of testing N(0,1) because we scaled by the standard deviation and shifted by the mean of each simulation scenario for meaningful comparisons.

The simulation steps can be made more understandable by showing them with a schematic diagram.

Response 16: Thank you for your suggestion. We thoroughly explained all simulation steps in Section 2.2. [Lines 130-153] (Such a thorough explanation with a schematic diagram would be challenging, so we decided to write all in complete sentences. We hope our intention is understandable.)

It is useful to give the power of the test in tables as well as graphs.

Response 17: We believe that this specific comment is associated with the general comment which is responded to in Response 5. (Power is not an appropriate criterion in parameter estimation.)

Discussion

Discussion of the results of the study in this section is rather poorly. It's more like the conclusion part. This section needs a review as there are only 8 literatures in this section.

Response 18: Given the length of the current manuscript, we tried to be concise in the discussion section (Section 6). We focused on the summary and benefits of the optimal and adaptive designs discussed in the manuscript. In Section 6, we tried to avoid lengthy elaborations and limitations which are already discussed in the subsections: Sections 2.3, 4.5, and 5.4. If you believe that there are other important points which will benefit readers in Section 6, we will be happy to expand the section.

Conclusion

This part does not exist. What is the take home message from the current study? This should be addressing the main aim (also the topic).

Response 19: Thank you for your suggestion. Section 7 (Conclusion) is added to the revised manuscript. [Lines 481-490]

Reference

T ASLAN, E., KOŞKAN, Ö., & ALTAY, Y. (2021). Determination of the Sample Size on Different Independent K Group Comparisons by Power Analysis. Türkiye Tarımsal Araştırmalar Dergisi, 8(1), 34-41.

Response 20: We are uncertain whether this reference is related to any part of the manuscript. This reference focuses on the determination of the sample size. Our paper focuses on the determination of distributing a fixed sample size to improve statistical inference. If we misunderstood your intention of listing this reference, please let us know.

Figure A. Power comparisons between the balanced design (dashed) and the adaptive design (solid) under the violation of normality assumption: T(10), T(5), Beta(5, 10), Beta(10, 5), and ^{2}(3).

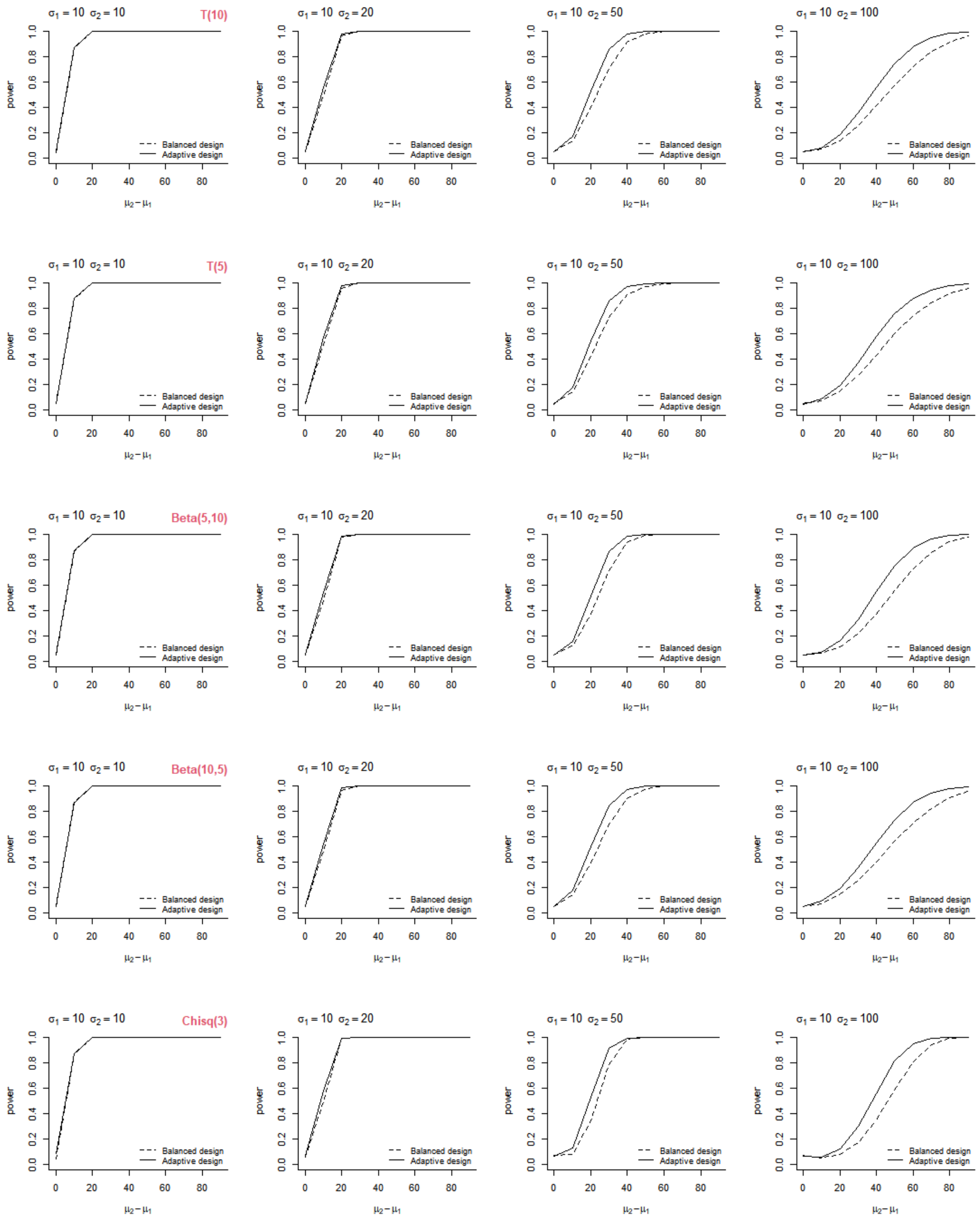

Supplement: Attachment — Submitted filename: Response to Reviewers 2021-08-17.pdf [file pone.0257472.s004.pdf]
